# *trans*-Tetra­kis(4-methyl­pyridine-κ*N*)dioxidorhenium(V) hexa­fluorido­phosphate

**DOI:** 10.1107/S160053681002458X

**Published:** 2010-06-26

**Authors:** Takeshi Kawasaki, Ali Canlier, Shubhamoy Chowdhury, Yasuhisa Ikeda

**Affiliations:** a Research Laboratory for Nuclear Reactors, Tokyo Institute of Technology, 2-12-1-N1-34 Ookayama, Meguro-ku, Tokyo 152-8550, Japan

## Abstract

The title compound, [ReO_2_(C_6_H_7_N)_4_]PF_6_, contains octa­hedral [ReO_2_(4-Mepy)_4_]^+^ cations (4-Mepy is 4-methyl­pyridine) and PF_6_^−^ anions. Both the cation and the anion reside on special positions, the Re atom on a crystallographic center of inversion and the P atom on a *C*_2_ axis parallel to the *b* axis. The Re^V^ atom in the cation exhibits an octa­hedral coordination geometry with two O atoms in the apical positions and four N atoms of the 4-Mepy ligands in the equatorial plane. The Re=O and Re—N bond lengths fall in the typical ranges of *trans*-dioxidorhenium(V) complexes.

## Related literature

For rhenium(V) complexes as radiopharmaceuticals, see: Dilworth & Parrott (1998 ▶); Volkert & Hoffman (1999 ▶). *trans-*Dioxidorhenium(V) ReO_2_^+^ complexes exhibit inter­esting properties as redox- and photo-active catalysts, see: Grey *et al.* (2004 ▶); Pipes & Meyer (1985 ▶); Thorp *et al.* (1989 ▶). For the synthesis of the title compound, see: Brewer & Gray (1989 ▶). For the crystal structures of *trans*-dioxidorhenium(V) complexes, see: Bélanger & Beauchamp (1996 ▶); Canlier *et al.* (2010 ▶); Gancheff *et al.* (2006 ▶); Kochel (2006 ▶); Kremer *et al.* (1996 ▶); Machura *et al.* (2008 ▶); Luck & O’Neill (2001 ▶); Reddy *et al.* (1999 ▶); Siczek *et al.* (2009 ▶).
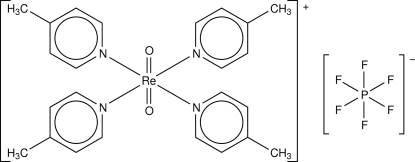


## Experimental

### 

#### Crystal data

[ReO_2_(C_6_H_7_N)_4_]PF_6_
*M* *_r_* = 735.68
Monoclinic, 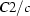

*a* = 10.4914 (4) Å
*b* = 19.5359 (8) Å
*c* = 14.0923 (5) Å
β = 109.5810 (11)°
*V* = 2721.31 (18) Å^3^
*Z* = 4
Mo *K*α radiation
μ = 4.60 mm^−1^
*T* = 173 K
0.26 × 0.13 × 0.12 mm

#### Data collection

Rigaku R-AXIS RAPID diffractometer
Absorption correction: multi-scan (*ABSCOR*; Higashi, 1995 ▶) *T*_min_ = 0.354, *T*_max_ = 0.576
12743 measured reflections
3113 independent reflections
2556 reflections with *F* ^2^ > 2σ(*F* ^2^)
*R*_int_ = 0.021

#### Refinement

*R*[*F* ^2^ > 2σ(*F* ^2^)] = 0.022
*wR*(*F* ^2^) = 0.044
*S* = 1.16
3113 reflections
175 parameters
H-atom parameters constrained
Δρ_max_ = 0.46 e Å^−3^
Δρ_min_ = −0.39 e Å^−3^

### 

Data collection: *PROCESS-AUTO* (Rigaku, 2006 ▶); cell refinement: *PROCESS-AUTO*; data reduction: *CrystalStructure* (Rigaku/MSC, 2006 ▶); program(s) used to solve structure: *SIR92* (Altomare *et al.*, 1994 ▶); program(s) used to refine structure: *SHELXL97* (Sheldrick, 2008 ▶); molecular graphics: *CrystalMaker* (CrystalMaker, 2007 ▶); software used to prepare material for publication: *CrystalStructure* (Rigaku/MSC, 2006 ▶).

## Supplementary Material

Crystal structure: contains datablocks global, I. DOI: 10.1107/S160053681002458X/zq2045sup1.cif

Structure factors: contains datablocks I. DOI: 10.1107/S160053681002458X/zq2045Isup2.hkl

Additional supplementary materials:  crystallographic information; 3D view; checkCIF report

## Figures and Tables

**Table:** 

Re1—O1	1.7688 (19)
Re1—N1	2.147 (2)
Re1—N2	2.146 (2)

**Table:** 

O1—Re1—O1^i^	180.00 (12)
O1—Re1—N1	90.33 (9)
O1—Re1—N2	90.35 (8)
N1—Re1—N2	90.20 (8)

Symmetry code: (i) 
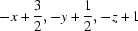
.

**Fig. 1.**
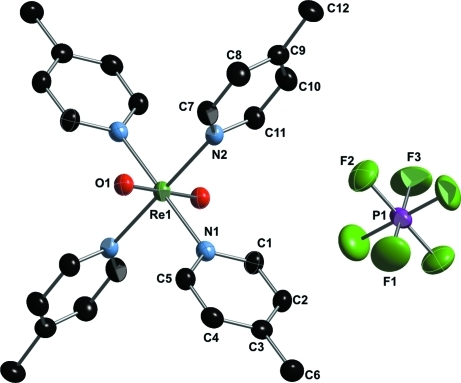
View of the [ReO2(4-Mepy)4]+ cation and PF6- anion with 50% thermal ellipsoids. Hydrogen atoms are omitted clarity.

**Fig. 2.**
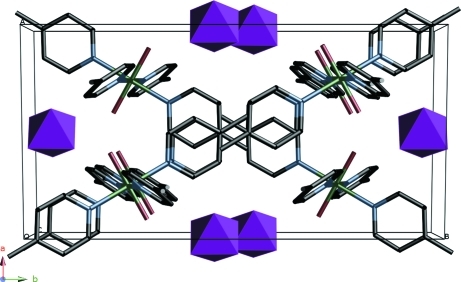
Packing view of the [ReO2(4-Mepy)4]+ (stick) and PF6- (octahedron) along the c axis.

## supplementary crystallographic information

### Comment

Rhenium(V) complexes as radiopharmaceuticals for therapy and diagnosis continue to attract attention, because rhenium isotopes have suitable radionuclear properties for the applications, *i.e.*, ^186^Re: *E*_max_ = 1.1 MeV for β-emission and *E*_max_ = 0.137 MeV for γ-emission with *t*_1/2_ = 90.6 h, ^188^Re: *E*_max_ = 2.1 MeV for β-emission and *E*_max_ = 0.155 MeV for γ-emission with *t*_1/2_ = 17 h (Dilworth & Parrott, 1998; Volkert & Hoffman, 1999). On the other hand, *trans-*dioxorhenium(V) ReO_2_^+^ complexes have been known to exhibit interesting properties as redox- and photo-active catalysts (Grey *et al.*, 2004; Pipes & Meyer, 1985; Thorp *et al.*,1989). To our knowledge, the title compound of formula [ReO_2_(4-Mepy)_4_]^+^.PF_6_^-^ (4-Mepy = 4-methylpyridine) (I) was already synthesized by Brewer & Gray (Brewer & Gray, 1989), but a crystallographic characterization has not been yet reported. In this article, we report the X-ray crystal structure of the title compound.

Complex I crystallized in the centrosymmetric space group *C*2/*c*. The crystal structure is constructed by the packing of [ReO_2_(4-Mepy)_4_]^+^ cations and octahedral PF_6_^-^ anions as shown Figs. 1 and 2. The Re atom is located on a crystallographic center of inversion and the P atom lies on a *C*_2_ axis parallel to the *b* axis. The Re^V^ atom in the cation exhibits an octahedral coordination geometry with two O atoms in the apical positions and four N atoms of the 4-Mepy ligands in the equatorial plane. The Re═O and Re—N bond lengths fall in the typical ranges of *trans*-dioxorhenium(V) complexes. No classical hydrogen bonds are observed in the crystal structure. The N1—Re1—N2 bond angle and all N—Re1═O angles are almost 90 °. The bond lengths of Re1—O1N, Re1—N1, and Re1—N2 are 1.769 (2), 2.147 (2) and 2.146 (2) Å, respectively. These values are similar to those found for other *trans*-dioxorhenium(V) complexes (Bélanger & Beauchamp, 1996; Canlier *et al.*, 2010; Gancheff *et al.*, 2006; Kochel, 2006; Kremer *et al.*, 1996; Machura *et al.*, 2008; Luck & O'Neill, 2001; Reddy *et al.*, 1999; Siczek *et al.*, 2009).

### Experimental

The title complex was synthesized according to the literature method by Brewer & Gray (Brewer & Gray, 1989). [ReO_2_(PPh_3_)_2_]I was reacted with 4-Mepy in methanol and the resulting [ReO_2_(4-Mepy)_4_]I was reacted with NH_4_PF_6_ in methanol.

### Refinement

All H atoms were positionated geometrically, with C—H = 0.95 and 0.98 Å for aromatic and methyl H atoms, respectively, and constrained to ride on their parent atoms, with *U*_iso_(H) = 1.2*U*_eq_(C).

### Crystal data

**Table:**

[ReO_2_(C_6_H_7_N)_4_]PF_6_	*F*(000) = 1440.00
*M**_r_* = 735.68	*D*_x_ = 1.796 Mg m^−^^3^
Monoclinic, *C*2/*c*	Mo *K*α radiation, λ = 0.71075 Å
Hall symbol: -C 2yc	Cell parameters from 13289 reflections
*a* = 10.4914 (4) Å	θ = 3.1–27.4°
*b* = 19.5359 (8) Å	µ = 4.60 mm^−^^1^
*c* = 14.0923 (5) Å	*T* = 173 K
β = 109.5810 (11)°	Block, orange
*V* = 2721.31 (18) Å^3^	0.26 × 0.13 × 0.12 mm
*Z* = 4	

### Data collection

**Table:**

Rigaku R-AXIS RAPID diffractometer	3113 independent reflections
Radiation source: fine-focus sealed tube	2556 reflections with *F*^2^ > 2σ(*F*^2^)
graphite	*R*_int_ = 0.021
Detector resolution: 10.00 pixels mm^-1^	θ_max_ = 27.4°
ω scans	*h* = −12→13
Absorption correction: multi-scan (*ABSCOR*; Higashi, 1995)	*k* = −25→25
*T*_min_ = 0.354, *T*_max_ = 0.576	*l* = −18→18
12743 measured reflections	

### Refinement

**Table:**

Refinement on *F*^2^	Secondary atom site location: difference Fourier map
*R*[*F*^2^ > 2σ(*F*^2^)] = 0.022	Hydrogen site location: inferred from neighbouring sites
*wR*(*F*^2^) = 0.044	H-atom parameters constrained
*S* = 1.16	*w* = 1/[σ^2^(*F*_o_^2^) + (0.0157*P*)^2^ + 6.0203*P*] where *P* = (*F*_o_^2^ + 2*F*_c_^2^)/3
3113 reflections	(Δ/σ)_max_ < 0.001
175 parameters	Δρ_max_ = 0.46 e Å^−^^3^
0 restraints	Δρ_min_ = −0.39 e Å^−^^3^
Primary atom site location: structure-invariant direct methods	

### Special details

**Table:**

Geometry. All e.s.d.'s (except the e.s.d. in the dihedral angle between two l.s. planes) are estimated using the full covariance matrix. The cell e.s.d.'s are taken into account individually in the estimation of e.s.d.'s in distances, angles and torsion angles; correlations between e.s.d.'s in cell parameters are only used when they are defined by crystal symmetry. An approximate (isotropic) treatment of cell e.s.d.'s is used for estimating e.s.d.'s involving l.s. planes.
Refinement. Refinement of *F*^2^ against ALL reflections. The weighted *R*-factor *wR* and goodness of fit *S* are based on *F*^2^, conventional *R*-factors *R* are based on *F*, with *F* set to zero for negative *F*^2^. The threshold expression of *F*^2^ > σ(*F*^2^) is used only for calculating *R*-factors(gt) *etc*. and is not relevant to the choice of reflections for refinement. *R*-factors based on *F*^2^ are statistically about twice as large as those based on *F*, and *R*- factors based on ALL data will be even larger.

### Fractional atomic coordinates and isotropic or equivalent isotropic displacement parameters (Å^2^)

**Table:**

	*x*	*y*	*z*	*U*_iso_*/*U*_eq_	
Re1	0.7500	0.2500	0.5000	0.02256 (4)	
P1	0.5000	0.04791 (6)	0.7500	0.0334 (2)	
F1	0.5776 (2)	0.10485 (15)	0.8276 (2)	0.0838 (8)	
F2	0.6185 (2)	0.04807 (14)	0.70289 (19)	0.0670 (6)	
F3	0.4238 (3)	−0.00954 (16)	0.6730 (2)	0.0962 (10)	
O1	0.90491 (19)	0.29340 (10)	0.52054 (14)	0.0268 (4)	
N1	0.7527 (2)	0.27819 (12)	0.64789 (17)	0.0257 (4)	
N2	0.8575 (2)	0.15747 (12)	0.55935 (16)	0.0249 (4)	
C1	0.6844 (3)	0.24197 (16)	0.6970 (2)	0.0311 (6)	
C2	0.6861 (3)	0.25974 (15)	0.7926 (2)	0.0331 (6)	
C3	0.7583 (3)	0.31654 (15)	0.8417 (2)	0.0311 (6)	
C4	0.8272 (3)	0.35354 (15)	0.7905 (2)	0.0319 (6)	
C5	0.8225 (2)	0.33362 (15)	0.6953 (2)	0.0294 (5)	
C6	0.7652 (3)	0.33635 (18)	0.9466 (2)	0.0440 (8)	
C7	0.9942 (3)	0.15588 (16)	0.5950 (2)	0.0331 (6)	
C8	1.0667 (3)	0.09651 (17)	0.6268 (2)	0.0372 (6)	
C9	1.0011 (3)	0.03451 (16)	0.6232 (2)	0.0323 (6)	
C10	0.8602 (3)	0.03686 (16)	0.5894 (2)	0.0365 (6)	
C11	0.7926 (3)	0.09779 (15)	0.5586 (2)	0.0313 (6)	
C12	1.0782 (3)	−0.03129 (17)	0.6533 (2)	0.0418 (7)	
H1	0.6337	0.2031	0.6650	0.037*	
H2	0.6373	0.2328	0.8249	0.040*	
H4	0.8780	0.3928	0.8209	0.038*	
H5	0.8704	0.3600	0.6617	0.035*	
H6A	0.7107	0.3044	0.9706	0.053*	
H6B	0.7299	0.3829	0.9458	0.053*	
H6C	0.8594	0.3347	0.9916	0.053*	
H7	1.0423	0.1975	0.5982	0.040*	
H8	1.1628	0.0981	0.6514	0.045*	
H10	0.8102	−0.0040	0.5876	0.044*	
H11	0.6965	0.0977	0.5360	0.038*	
H12A	1.0144	−0.0693	0.6453	0.050*	
H12B	1.1370	−0.0284	0.7238	0.050*	
H12C	1.1334	−0.0391	0.6103	0.050*	

### Atomic displacement parameters (Å^2^)

**Table:**

	*U*^11^	*U*^22^	*U*^33^	*U*^12^	*U*^13^	*U*^23^
Re1	0.01850 (7)	0.02494 (7)	0.02294 (7)	−0.00157 (8)	0.00521 (5)	0.00297 (7)
P1	0.0302 (5)	0.0239 (5)	0.0474 (6)	0.0000	0.0145 (4)	0.0000
F1	0.0809 (19)	0.0820 (19)	0.0810 (18)	−0.0331 (15)	0.0174 (14)	−0.0387 (15)
F2	0.0484 (13)	0.0881 (18)	0.0769 (15)	−0.0047 (12)	0.0375 (12)	0.0053 (14)
F3	0.095 (2)	0.084 (2)	0.127 (2)	−0.0475 (17)	0.0597 (19)	−0.0657 (18)
O1	0.0204 (9)	0.0301 (10)	0.0288 (9)	−0.0027 (7)	0.0066 (7)	0.0020 (8)
N1	0.0213 (11)	0.0257 (10)	0.0284 (11)	0.0004 (9)	0.0062 (9)	0.0022 (9)
N2	0.0233 (11)	0.0277 (11)	0.0229 (10)	−0.0007 (9)	0.0065 (8)	0.0027 (8)
C1	0.0310 (13)	0.0327 (16)	0.0304 (13)	−0.0047 (12)	0.0110 (10)	0.0022 (12)
C2	0.0357 (15)	0.0348 (18)	0.0317 (13)	0.0025 (12)	0.0150 (11)	0.0065 (12)
C3	0.0330 (15)	0.0319 (15)	0.0272 (13)	0.0116 (12)	0.0084 (11)	0.0046 (11)
C4	0.0310 (15)	0.0281 (14)	0.0333 (14)	0.0021 (12)	0.0062 (11)	−0.0013 (11)
C5	0.0275 (14)	0.0303 (14)	0.0295 (13)	−0.0015 (11)	0.0084 (11)	0.0029 (11)
C6	0.063 (2)	0.0379 (17)	0.0343 (16)	0.0082 (16)	0.0204 (15)	0.0007 (13)
C7	0.0248 (14)	0.0338 (15)	0.0378 (15)	−0.0013 (12)	0.0066 (11)	0.0063 (12)
C8	0.0242 (14)	0.0424 (17)	0.0413 (16)	0.0057 (13)	0.0062 (12)	0.0076 (13)
C9	0.0362 (16)	0.0335 (15)	0.0267 (13)	0.0085 (12)	0.0099 (11)	0.0030 (11)
C10	0.0362 (16)	0.0288 (15)	0.0425 (16)	−0.0022 (13)	0.0106 (13)	0.0028 (12)
C11	0.0250 (14)	0.0326 (15)	0.0344 (15)	−0.0008 (12)	0.0073 (11)	0.0039 (12)
C12	0.0445 (19)	0.0348 (17)	0.0448 (18)	0.0137 (14)	0.0135 (14)	0.0044 (14)

### Geometric parameters (Å, °)

**Table:**

Re1—O1	1.7688 (19)	C4—C5	1.382 (4)
Re1—O1^i^	1.7688 (19)	C7—C8	1.377 (4)
Re1—N1	2.147 (2)	C8—C9	1.386 (4)
Re1—N1^i^	2.147 (2)	C9—C10	1.394 (4)
Re1—N2	2.146 (2)	C9—C12	1.502 (4)
Re1—N2^i^	2.146 (2)	C10—C11	1.379 (4)
P1—F1	1.581 (2)	C1—H1	0.950
P1—F1^ii^	1.581 (2)	C2—H2	0.950
P1—F2	1.593 (2)	C4—H4	0.950
P1—F2^ii^	1.593 (2)	C5—H5	0.950
P1—F3	1.579 (3)	C6—H6A	0.980
P1—F3^ii^	1.579 (3)	C6—H6B	0.980
N1—C1	1.351 (4)	C6—H6C	0.980
N1—C5	1.352 (3)	C7—H7	0.950
N2—C7	1.352 (3)	C8—H8	0.950
N2—C11	1.348 (3)	C10—H10	0.950
C1—C2	1.386 (4)	C11—H11	0.950
C2—C3	1.390 (3)	C12—H12A	0.980
C3—C4	1.384 (4)	C12—H12B	0.980
C3—C6	1.506 (4)	C12—H12C	0.980
			
O1—Re1—O1^i^	180.00 (12)	C2—C3—C6	122.2 (3)
O1—Re1—N1	90.33 (9)	C4—C3—C6	121.1 (2)
O1—Re1—N1^i^	89.67 (9)	C3—C4—C5	120.3 (2)
O1—Re1—N2	90.35 (8)	N1—C5—C4	122.9 (2)
O1—Re1—N2^i^	89.65 (8)	N2—C7—C8	122.8 (2)
O1^i^—Re1—N1	89.67 (9)	C7—C8—C9	120.7 (2)
O1^i^—Re1—N1^i^	90.33 (9)	C8—C9—C10	116.2 (2)
O1^i^—Re1—N2	89.65 (8)	C8—C9—C12	121.6 (2)
O1^i^—Re1—N2^i^	90.35 (8)	C10—C9—C12	122.2 (2)
N1—Re1—N1^i^	180.00 (12)	C9—C10—C11	120.7 (2)
N1—Re1—N2	90.20 (8)	N2—C11—C10	122.6 (2)
N1—Re1—N2^i^	89.80 (8)	N1—C1—H1	119.0
N1^i^—Re1—N2	89.80 (8)	C2—C1—H1	119.0
N1^i^—Re1—N2^i^	90.20 (8)	C1—C2—H2	119.6
N2—Re1—N2^i^	180.00 (12)	C3—C2—H2	119.6
F1—P1—F1^ii^	90.54 (15)	C3—C4—H4	119.9
F1—P1—F2	89.65 (15)	C5—C4—H4	119.9
F1—P1—F2^ii^	90.19 (15)	N1—C5—H5	118.6
F1—P1—F3	179.37 (16)	C4—C5—H5	118.6
F1—P1—F3^ii^	90.02 (14)	C3—C6—H6A	109.5
F1^ii^—P1—F2	90.19 (15)	C3—C6—H6B	109.5
F1^ii^—P1—F2^ii^	89.65 (15)	C3—C6—H6C	109.5
F1^ii^—P1—F3	90.02 (14)	H6A—C6—H6B	109.5
FP^ii^—P1—F3^ii^	179.37 (14)	H6A—C6—H6C	109.5
F2—P1—F2^ii^	179.78 (16)	H6B—C6—H6C	109.5
F2—P1—F3	90.06 (16)	N2—C7—H7	118.6
F2—P1—F3^ii^	90.10 (16)	C8—C7—H7	118.6
F2^ii^—P1—F3	90.10 (16)	C7—C8—H8	119.6
F2^ii^—P1—F3^ii^	90.06 (16)	C9—C8—H8	119.6
F3—P1—F3^ii^	89.41 (16)	C9—C10—H10	119.6
Re1—N1—C1	121.74 (18)	C11—C10—H10	119.6
Re1—N1—C5	120.9 (2)	N2—C11—H11	118.7
C1—N1—C5	117.3 (2)	C10—C11—H11	118.7
Re1—N2—C7	121.15 (19)	C9—C12—H12A	109.5
Re1—N2—C11	121.84 (18)	C9—C12—H12B	109.5
C7—N2—C11	116.9 (2)	C9—C12—H12C	109.5
N1—C1—C2	122.1 (2)	H12A—C12—H12B	109.5
C1—C2—C3	120.8 (3)	H12A—C12—H12C	109.5
C2—C3—C4	116.7 (2)	H12B—C12—H12C	109.5

Symmetry codes: (i) −*x*+3/2, −*y*+1/2, −*z*+1; (ii) −*x*+1, *y*, −*z*+3/2.
